# Comparison of the efficacy and safety of picosecond Nd:YAG laser (1,064 nm), picosecond alexandrite laser (755 nm) and 2% hydroquinone cream in the treatment of melasma: A randomized, controlled, assessor-blinded trial

**DOI:** 10.3389/fmed.2023.1132823

**Published:** 2023-03-28

**Authors:** Surong Liang, Shuai Shang, Wensi Zhang, Ansheng Tan, Boyang Zhou, Xueling Mei, Linfeng Li

**Affiliations:** Department of Dermatology, Beijing Friendship Hospital, Capital Medical University, Being, China

**Keywords:** melasma, picosecond laser, picosecond Nd:YAG laser, picosecond alexandrite laser, hydroquinone

## Abstract

**Background:**

Increasing numbers of studies demonstrated that picosecond lasers (Picos) were effective and safe for melasma. However, A limited number of randomized controlled trials (RCTs) regarding Picos contribute to a modest level of evidence. Topical hydroquinone (HQ) remains to be the first-line therapy.

**Objective:**

To compare the efficacy and safety of non-fractional picosecond Nd:YAG laser (PSNYL), non-fractional picosecond alexandrite laser (PSAL), and 2% HQ cream in the treatment of melasma.

**Method:**

Sixty melasma patients with Fitzpatrick skin types (FST) III-IV were randomly assigned to the PSNY, PSAL, and HQ groups at a 1:1:1 ratio. Patients in PSNYL and PSAL groups received 3 laser sessions at 4-week intervals. The 2% HQ cream was applied twice daily for 12 weeks in patients of the HQ group. The primary outcome, the melasma area and severity index (MASI) score, was evaluated at weeks 0, 4, 8, 12, 16, 20, and 24. The patient assessment score by quartile rating scale was rated at weeks 12, 16, 20, and 24.

**Results:**

Fifty-nine (98.3%) subjects were included in the analysis. Each group showed significant change from baseline in MASI scores from week 4 to week 24. The MASI score in the PSNYL group showed the greatest reduction compared to the PSAL group (*p* = 0.016) and HQ group (*p* = 0.018). The PSAL group demonstrated comparable MASI improvement as the HQ group (*p* = 0.998). The PSNYL group had the highest patient assessment score, followed by the PSAL group and then the HQ group, although only the differences between PSNYL and HQ groups at weeks 12 and 16 were significant. Four patients (6.8%) experienced recurrence. Other unanticipated events were transient and subsided after 1 week to 6 months.

**Conclusion:**

The efficacy of non-fractional PSNYL was superior to that of non-fractional PSAL, which was not inferior to 2% HQ, thus non-fractional Picos providing an alternative for melasma patients with FSTs III-IV. The safety profiles of PSNYL, PSAL, and 2% HQ cream were similar.

**Clinical Trial Registration:**

https://www.chictr.org.cn/showprojen.aspx?proj=130994, ChiCTR2100050089.

## Introduction

Melasma is a disfiguring pigmentary condition that typically affects the face in females with darker skin types, such as Fitzpatrick skin types (FST) III and IV (1), which is psychosocially harmful to affected patients. The prevalence ranges from 8.8–40% in high-risk populations (1). The general feature of the pathology is hyperactive melanocytes containing more melanosomes shown in the epidermis. Topical hydroquinone (HQ) remains the first-line therapy for melasma. It can reduce melanogenesis by interfering with the activity of tyrosinase and alter the formation of melanosomes by inhibiting RNA and DNA synthesis (2). As a result of these properties, melanocyte metabolism is suppressed, resulting in a gradual decrease in melanin production. HQ cream is typically used at a concentration of 2–5%, but the most available concentration in mainland China is 2%. It can be applied twice a day at the beginning of therapy and pigment lightening can be observed after 5–8 weeks. Adverse reactions, like local irritation, hyperpigmentation, and hypopigmentation, can occur. And some patients fail to achieve the desired depigmenting effect as they cannot adhere to daily use at first, so they often seek other treatment options, such as laser and light therapy.

For more than a decade, lasers have been studied for melasma treatment and large-spot and low-fluence Q-switched Nd:YAG 1064 nm laser (QSNYL) has been widely used and proved effective (3). Recently, a systematic review and network meta-analysis which selected 59 randomized controlled trials (RCTs) to compare the efficacy and side effects of 14 common therapies for melasma showed that QSNYL ranked first in efficacy and was worthy of being used as a monotherapy or in combination therapy for melasma (4). The main mechanism of QSNYL is subcellular selective photothermolysis, which means that the laser energy-induced photothermal effect targets melanosomes and shatters them into tiny particles, thus facilitating the clearance of melanin particles by phagocytes (2, 3). However, the thermal effect could cause damage and inflammation to surrounding tissues which may result in a high incidence of side effects such as post-inflammatory hyperpigmentation (PIH) and hypopigmentation (5).

Picosecond lasers (Picos), a kind of newly emerging lasers with an extraordinarily short pulse duration of 300–900 picoseconds applied in dermatology (6), produce a more photomechanical than photothermal effect, which causes pigment fragmentation more efficiently while minimizing thermal damage to the surrounding tissue (7). Picos prescribed for melasma mainly included 1,064 nm picosecond Nd:YAG laser (PSNYL) and 755 nm picosecond alexandrite laser (PSAL). It has been shown that non-fractional PSAL achieved a better and faster clearance rate for melasma compared with QSNYL (8, 9). There is also a study indicating that fractional PSAL showed comparable efficacy with the triple combination cream (TCC) (10), a lightening agent containing 4% hydroquinone and considered the gold standard treatment of melasma. And the combination therapy of fractional PSNYL and 4% HQ was significantly better than 4% HQ alone (11). These results indicate an exciting prospect of Picos for melasma therapy. A limited number of RCTs on Picos, however, contribute to the modest level of evidence (3, 4). Furthermore, several questions remain unanswered including the effectiveness and tolerance of 755 nm versus 1,064 nm Picos, non-fractional Picos versus the standard topical treatments (HQ or TCC), and optimal treatment settings of non-fractional Picos (6).

The objective of this study was to compare the efficacy and safety of non-fractional PSNYL (1,064 nm), non-fractional PSAL (755 nm) and 2% HQ in melasma patients with FSTs III-IV.

## Materials and method

### Type of study

This was a prospective, randomized, controlled, accessor-blinded study conducted at a single center in Beijing between 15 August 2021 and 10 June 2022. The study was registered on the Chinese Clinical Trial Registry (ChiCTR2100050089) and approved by the Ethical Committee of Beijing Friendship Hospital, Capital Medical University (2021-P2-118-02). Written informed consent was obtained from all subjects before study entry.

### Patients

Recruitment took place between August 2021 and December 2021, during which 60 patients were recruited from a single tertiary skin center of a university hospital in Beijing. Inclusion criteria were female melasma patients, aged 18–65 years, and FSTs III--IV. Exclusion criteria were (1) pregnancy or lactation; (2) unable to follow strict sun-protective guidelines due to outside work; (3) contact allergy to HQ; (4) photosensitive conditions; (5) active stage of melasma; (6) treatment with laser, intense pulsed light, chemical peeling, oral bleaching agent or steroid hormones (e.g., corticosteroids, contraceptive pills) within the preceding 6 months; (7) treatment with topical HQ or other bleaching agents such as azelaic acid, ascorbic acid or topical retinoid within the preceding 4 weeks; (8) treatment with oral photosensitizing drugs (e.g., sulfonamides, tetracyclines) within the preceding 4 weeks.

### Randomization

**Figure 1:**
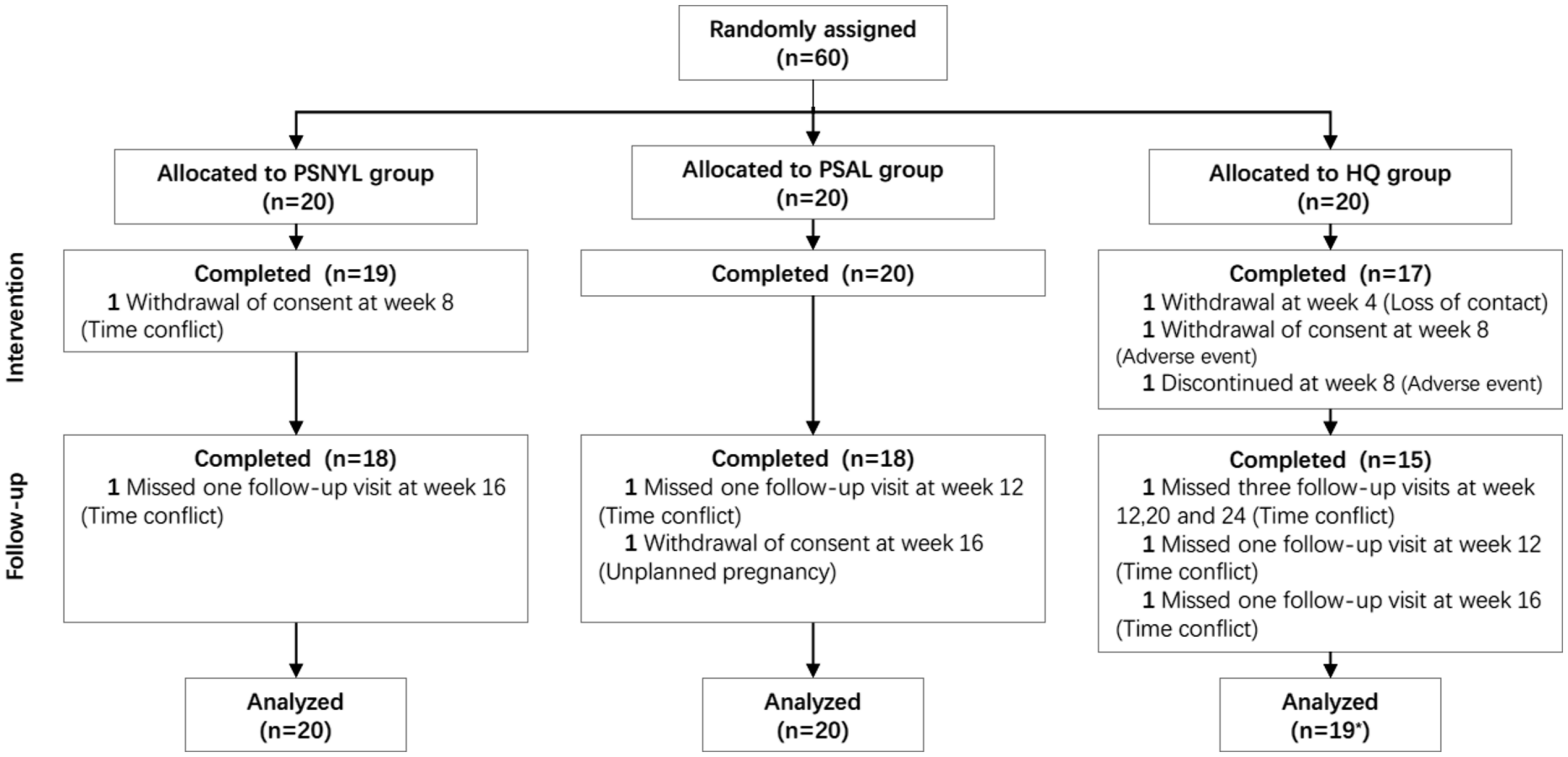
Flowchart of participants. PSNYL, picosecond Nd:YAG laser (1,064 nm); PSAL, picosecond alexandrite laser (755 nm); HQ, hydroquinone (2%). * One participant in the HQ group shed early during the intervention period (week 4), and thus was not included in the analysis.

Sixty eligible patients were randomized to 3 groups (PSNYL, PSAL, and HQ) in a 1:1:1 ratio by computer-generated random numbers (Figure 1). Because of the nature of the laser interventions, neither the participants nor the laser operator were unable to be blinded. However, assessors were blinded to the group allocation while evaluating study outcomes.

### Intervention

PSNYL group: A 1064 nm picosecond Nd:YAG laser (PSNYL) with a zoom handpiece (Picoway; Candela, Massachusetts, United States) was used and parameters were shown in Table 1.

**Table 1:** Laser treatment parameters.

Parameters	PSNYL group	PSAL group
Wavelength, nm	1,064	755
Pulse duration, ps	450	750
Mode	Non-fractioned	
Spot size, mm	7	6–8
Fluence, J/cm^2^	0.75–0.90	0.40–0.71
Repetition, Hz	8	10
No. of passes per treatment	2	2
Average pulses per treatment	4,500	4,100
Endpoint	Mild erythema	
No. of treatment sessions	3	
Interval	4-week	

PSNYL, picosecond Nd: YAG laser; PSAL, picosecond alexandrite laser.

PSAL group: A 755 nm picosecond alexandrite laser (PSAL) with a flat optic (Picosure; Cynosure, MA, United States) was used and parameters were shown in Table 1.

In both laser groups, two passes were delivered with one performed on the entire face and the other only on the pigmented areas. All patients in laser groups received a 20-min full-face cold spray of distilled water for cooling immediately after the laser treatment.

HQ group: Patients were asked to apply a 2% hydroquinone (HQ) cream (QianBai; REEKON, Guangdong, China) on the pigmented areas twice a day for 12 consecutive weeks.

Skincare and photoprotection: All subjects were instructed to continue their daily facial skincare but to avoid any topical skin-lighting products, chemical peels, or other laser procedures throughout the six-month study period. The importance of sun protection for the treatment success of melasma was highlighted when each of them received photoprotection education. They were asked to apply a broad-spectrum sunscreen with a sun protection factor (SPF) of 48, PA+++ (Winona; Botanee Bio-technology group CO., Yunnan, China) at 2 mg/cm^2^, which indicates one teaspoon for the face according to the teaspoon rule (12), at every 2 h during daylight. Besides applying sunscreen, multimodal photoprotection behaviors were encouraged while outdoors, such as avoiding sun exposure during peak hours, seeking shade, wearing sun-protective masks and hats, and using sunglasses and umbrellas.

### Outcome evaluation and follow-up

#### Primary outcome

The melasma area and severity index (MASI) score was the primary outcome. Two trained independent physicians were blinded to the group allocation of the patients. The physicians determined MASI scores based on the standardized photographs, which were collected from the front and both sides of the cheeks by using VISIA (Canfield Scientific, Parsippany, NJ) with the same background and a fixed angle. A higher MASI score refers to a greater severity and/or larger affected area of melasma.

**Figure 2:**
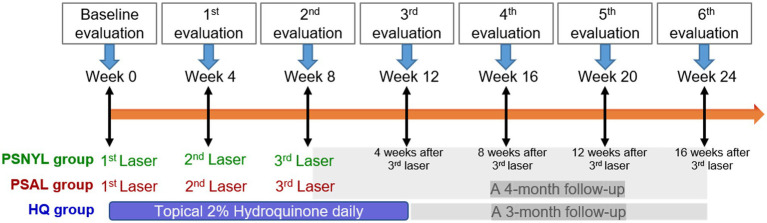
Study design. PSNYL, picosecond Nd:YAG laser (1,064 nm); PSAL, picosecond alexandrite laser (755 nm); HQ, hydroquinone (2%).

All three groups had MASI evaluations at week 4 (1 month after the first laser session), week 8 (1 month after the second laser session), week 12 (1 month after the third laser session), week 16 (2 months after the third laser session), week 20 (3 months after the third laser session), and week 24 (4 months after the third laser session). Therefore, at the time of the last evaluation at week 24, the PSNYL group and PSAL group had a 4-month follow-up and the HQ group had a 3-month follow-up (Figure 2).

#### Secondary outcome

The patient assessment score on the improvement of melasma was the secondary outcome. From week 12 to week 24, patients evaluated themselves on a quartile grading scale of 0 (no improvement or worsening), 1 (less than 25% improvement), 2 (25 to <50%improvement), 3 (50 to <75% improvement), and 4 (75 to 100% improvement).

#### Adverse or unanticipated events

Any adverse or unanticipated events related to the interventions were recorded during the six-month study, such as erythema and itch, hypopigmentation, post-inflammatory hyperpigmentation (PIH), and recurrence.

Patients in PSNYL and PSAL groups had additional pain level evaluation during the laser intervention period. They were asked to report pain severity using a visual analogue scale (VAS), which ranged from 0 (no pain) to 10 (most severe pain). And the duration of erythematous appearance after laser interventions were recorded.

### Statistical analysis

#### Sample size

No calculation was performed on sample size. The sample size was determined based on other clinical trials of melasma.

#### Analytical approach

The one-way analysis of variance (ANOVA), Kruskal-Wallis tests, and Fisher exact tests were used to evaluate between-group differences in baseline characteristics and adverse events among the three groups. Student’s *t*-test and Mann–Whitney U test were conducted to access between-group differences concerning adverse events between two laser groups.

Accounting for correlated repeated measurements and missing values, generalized estimating equations (GEE) were used to test within-group changes in MASI scores between each evaluation (week 4, 8, 12, 16, 20, or 24) and baseline (week 0), and patient rating scores between each evaluation (week 16, 20 or 24) and the first evaluation (week 12). GEE was also used to measure between-group differences for MASI scores and patient rating scores among the three groups. The patient assessment score was treated as a rank variable. In the analysis of between-group differences in MASI scores, group main effect, time main effect, and group-by-time interaction were included in the model, while the baseline MASI score was included as a covariate. A significant group-by-time interaction means that the effect of the intervention-mediated difference between groups changes over time (13). Group main effect indicates the effect of the intervention-mediated difference between groups, ignoring the effect of time. Differences between groups at individual timepoints were examined in GEE.

The Holm-Bonferroni correction was performed for multiple comparisons in MASI scores. Missing data were not filled in since GEE can provide an adequate account for these. A *p* value less than 0.05 was considered statistically significant. All statistical analyses were conducted using SPSS version 27.0 (IBM Corp, NY, United States).

## Results

### Patient characteristics

A total of 60 subjects were included in the study, and 85% (51/60) of subjects completed the protocol. Finally, 20, 20, and 19 subjects from PSNYL, PSAL, and HQ groups, respectively, were included in the analysis (Figure 1). One subject (1/60, 1.7%) in the HQ group shed early during the intervention period (after baseline evaluation) and was not included in the analysis. Baseline characteristics (age, disease duration, FST, and baseline MASI scores) did not differ statistically between groups (Table 2).

**Table 2:** Patients’ characteristics at baseline.

Characteristics	PSNYL group	PSAL group	HQ group	Between-group
	(*n* = 20)	(*n* = 20)	(*n* = 19)	*p*-value
Age, years, mean (SD)	37.8 (6.1)	39.5 (6.3)	41.8 (8.8)	0.211
Disease course, years, median (IQR)	5.5 (6.0)	7.0 (9.8)	8.0 (7.0)	0.767
Fitzpatrick skin type, *n* (%)				
Type III	5 (25.0)	4 (20.0)	5 (26.3)	0.930
Type IV	15 (75.0)	16 (80.0)	14 (73.7)	
Baseline MASI score, mean (SD)	15.6 (6.0)	14.9 (5.1)	16.7 (5.8)	0.581

PSNYL, picosecond Nd: YAG laser (1,064 nm); PSAL, picosecond alexandrite laser (755 nm); HQ, hydroquinone (2%); MASI, melasma area and severity index.

### Melasma area and severity index scores

**Figure 3:**
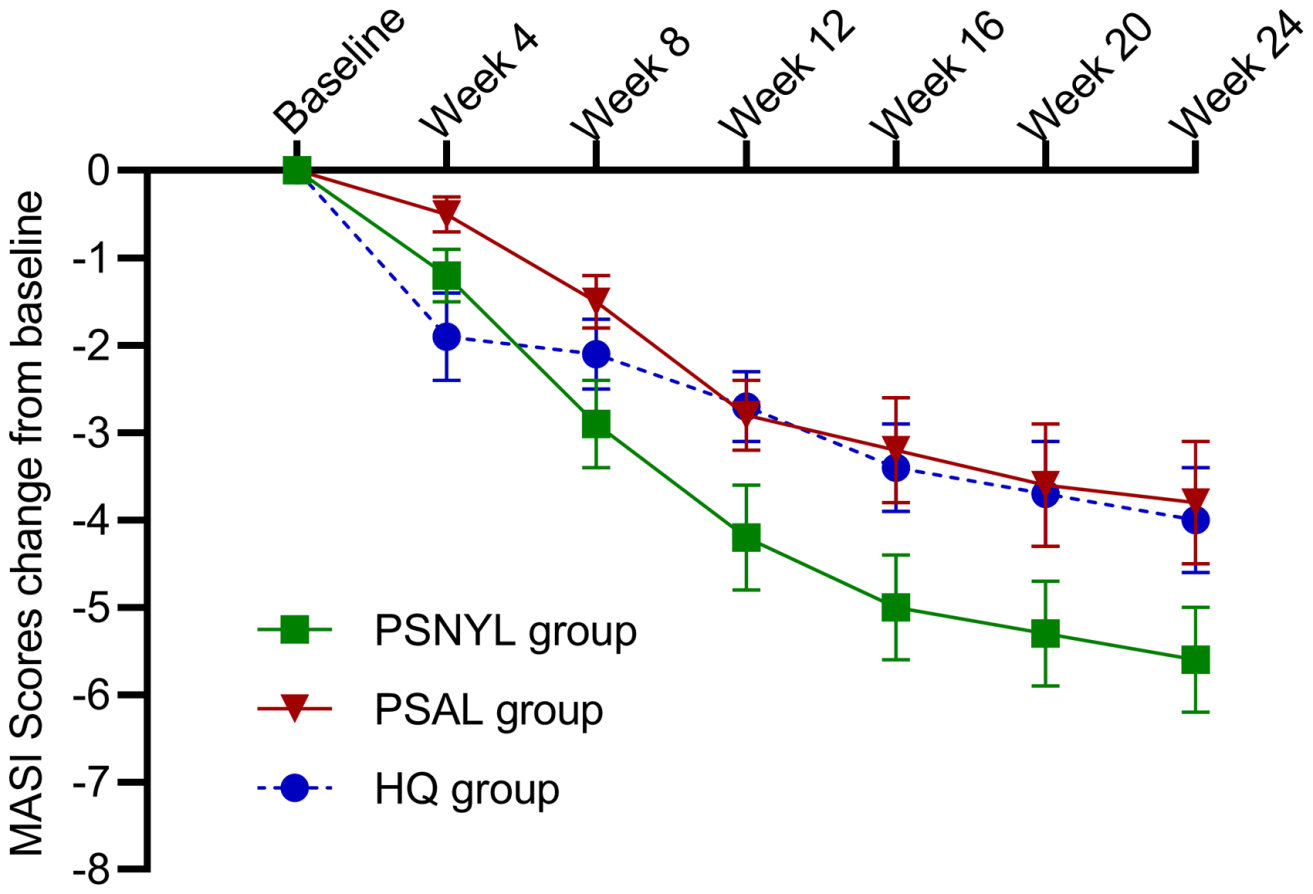
Change in MASI scores from baseline in the three groups. PSNYL, picosecond Nd:YAG laser (1,064 nm); PSAL, picosecond alexandrite laser (755 nm); HQ, hydroquinone (2%).

All three groups showed a trend toward improvement of the MASI score, even after the end of interventions (Figure 3). From week 4 to week 24, each group showed significant change from baseline in MASI scores at every timepoint (Table 3). At week 24 (4 months after the last laser session), the improvement rates were 35.9%, 25.5%, and 24.0% in groups PSNYL, PSAL, and HQ, respectively. The group-by-time interactive effect was not significant (*p* = 0.189), but the group main effect was significant for MASI scores between the three groups (*p* = 0.007), which suggested that different interventions contributed to the different outcomes of MASI scores between groups when ignoring the time effect. The MASI score in the PSNYL group showed the greatest improvement compared to the PSAL group (−1.3 [CI, −2.4 to −0.2]; *p* = 0.016) and HQ group (−1.3 [CI, −2.4 to −0.2]; *p* = 0.018). The PSAL group showed comparable improvement to the HQ group (0.001 [CI, −1.0 to 1.0]; *p* = 0.998).

**Table 3:** Outcomes of melasma area and severity index (MASI) scores.

Evaluation time	Within-group change from baseline							Between-group difference in MASI scores					
	PSNYL group		PSAL group		HQ group		Group main effect	PSNYL Group vs. PSAL Group		PSNYL group vs. HQ group		PSAL group vs. HQ group	
	(*n* = 20)		(*n* = 20)		(*n* = 19)								
	LS mean change (SE)	%	LS mean change (SE)	%	LS mean change (SE)	%		LS mean difference	*p* value	LS mean difference	*p* value	LS mean difference	*p* value
								(95% CI)		(95% CI)		(95% CI)	
Week 4	−1.2 (0.3)†	7.7	−0.5 (0.2)†	3.4	−1.9 (0.5)†	11.4	–	−0.5 (−1.5 to 0.4)	0.401	0.4 (−0.8 to 1.5)	0.516	0.9 (−0.2 to 2.1)	0.109
Week 8	−2.9 (0.5)†	18.6	−1.5 (0.3)†	10.1	−2.1 (0.4)†	12.6	–	−1.2 (−2.3 to −0.1)	**0.027**	−1.0 (−2.1 to 0.02)	0.055	0.2 (−0.8 to 1.1)	0.730
Week 12	−4.2 (0.6)†	26.9	−2.8 (0.4)†	18.8	−2.7 (0.4)†	16.2	–	−1.2 (−2.5 to 0.02)	0.055	−1.7 (−2.8 to −0.6)	**0.003**	−0.5 (−1.5 to 0.6)	0.366
Week 16	−5.0 (0.6)†	32.1	−3.2 (0.6)†	21.5	−3.4 (0.5)†	20.4	–	−1.6 (−3.2 to −0.1)	**0.040**	−1.9 (−3.2 to −0.6)	**0.003**	−0.3 (−1.6 to 1.0)	0.648
Week 20	−5.3 (0.6)†	34.0	−3.6 (0.7)†	24.2	−3.7 (0.6)†	22.2	–	−1.6 (−3.2 to −0.02)	**0.047**	−1.9 (−3.2 to −0.6)	**0.005**	−0.3 (−1.7 to 1.2)	0.705
Week 24	−5.6 (0.6)†	35.9	−3.8 (0.7)*	25.5	−4.0 (0.6)†	24.0	–	−1.6 (−3.2 to −0.04)	**0.044**	−1.9 (−3.1 to −0.6)	**0.003**	−0.2 (−1.6 to 1.1)	0.728
*p* value	<0.001	–	<0.001	–	<0.001	–	0.007	−1.3 (−2.4 to −0.2)	**0.016**	−1.3 (−2.4 to −0.2)	**0.018**	0.001 (−1.0 to 1.0)	0.998

PSNYL, picosecond Nd:YAG laser (1,064 nm); PSAL, picosecond alexandrite laser (755 nm); HQ, hydroquinone (2%); LS, least squares.

*p < 0·05 compared with baseline.

† *p* < 0·001 compared with baseline. *p* values in boldface indicate significance after the adjustment of the Holm-Bonferroni method for multiple comparisons.

The group main effect was examined by generalized estimating equations (GEE) with baseline MASI scores as the covariate. A significant group main effect suggested that different interventions contributed to the different outcomes of MASI scores among groups when ignoring the effect of time.

**Figure 4:**
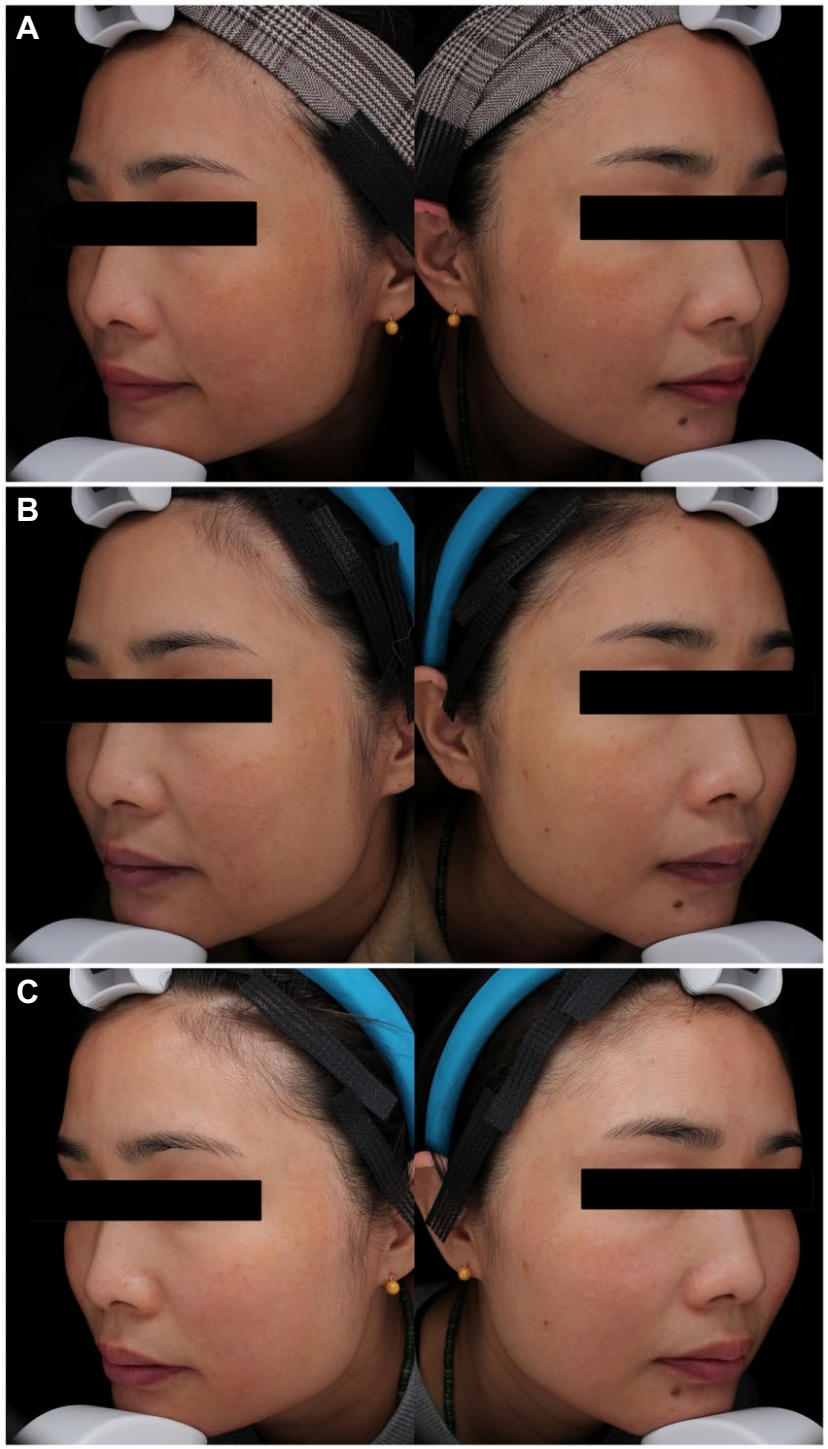
Melasma in a 41-year-old woman with Fitzpatrick skin type IV in the PSNYL (1,064 nm) group. **(A)** At baseline, MASI score = 18.6; **(B)** one month after three laser treatments (week 12), MASI score = 13.1; **(C)** four months after three laser treatments (week 24), MASI score = 11.7. PSNYL, picosecond Nd:YAG laser.

**Figure 5:**
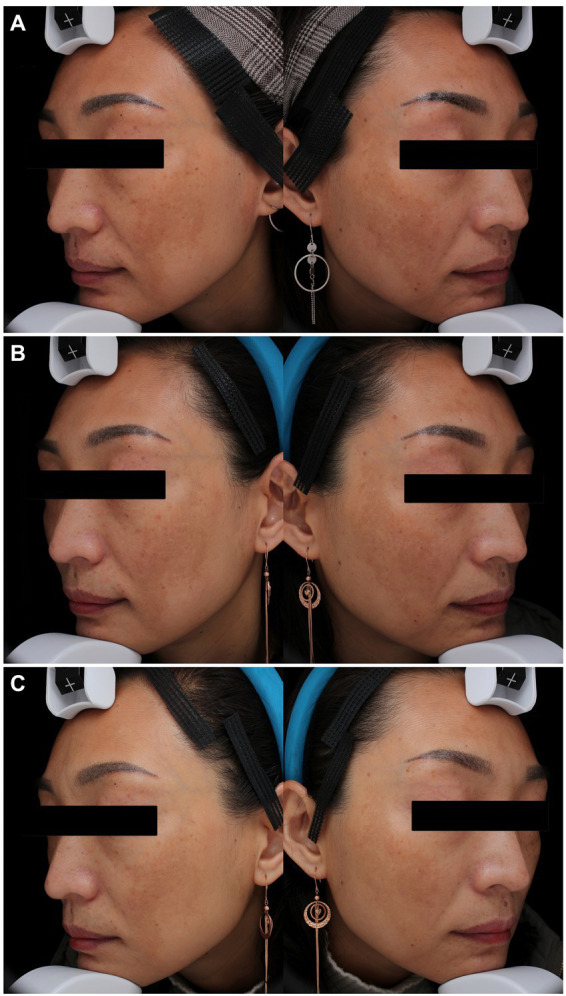
Melasma in a 43-year-old woman with Fitzpatrick skin type IV in the PSAL (755 nm) group. **(A)** At baseline, MASI score = 17.0; **(B)** one month after three laser treatments (week 12), MASI score = 15.6; **(C)** 4 months after three laser treatments (week 24), MASI score = 12.4. PSAL, picosecond alexandrite laser.

At week 4 (1 month after the 1^st^ laser session), the MASI score reduced more in the PSNYL group than in the PSAL group, while the HQ group showed the greatest reduction. But no significant differences were found between the three groups. At week 8 (1 month after the 2^nd^ laser session), the MASI score decreased most in the PSNYL group among the three groups, and there was a significant difference between the PSNYL group and the HQ group (*p* = 0.027). At week 12 (1 month after the 3^rd^ laser session), the MASI score in the PSNYL group still declined the most and showed a significant difference from the HQ group (*p* = 0.003). From week 16 to week 24 (2–4 months after the 3^rd^ laser session), the MASI score in the PSNYL group remained to reduce the most with significant differences from the PSAL group and HQ group (Table 3). The clinical photographs in groups PSNYL and PSAL are shown in Figures 4, 5, respectively.

### Patient assessment scores

**Figure 6:**
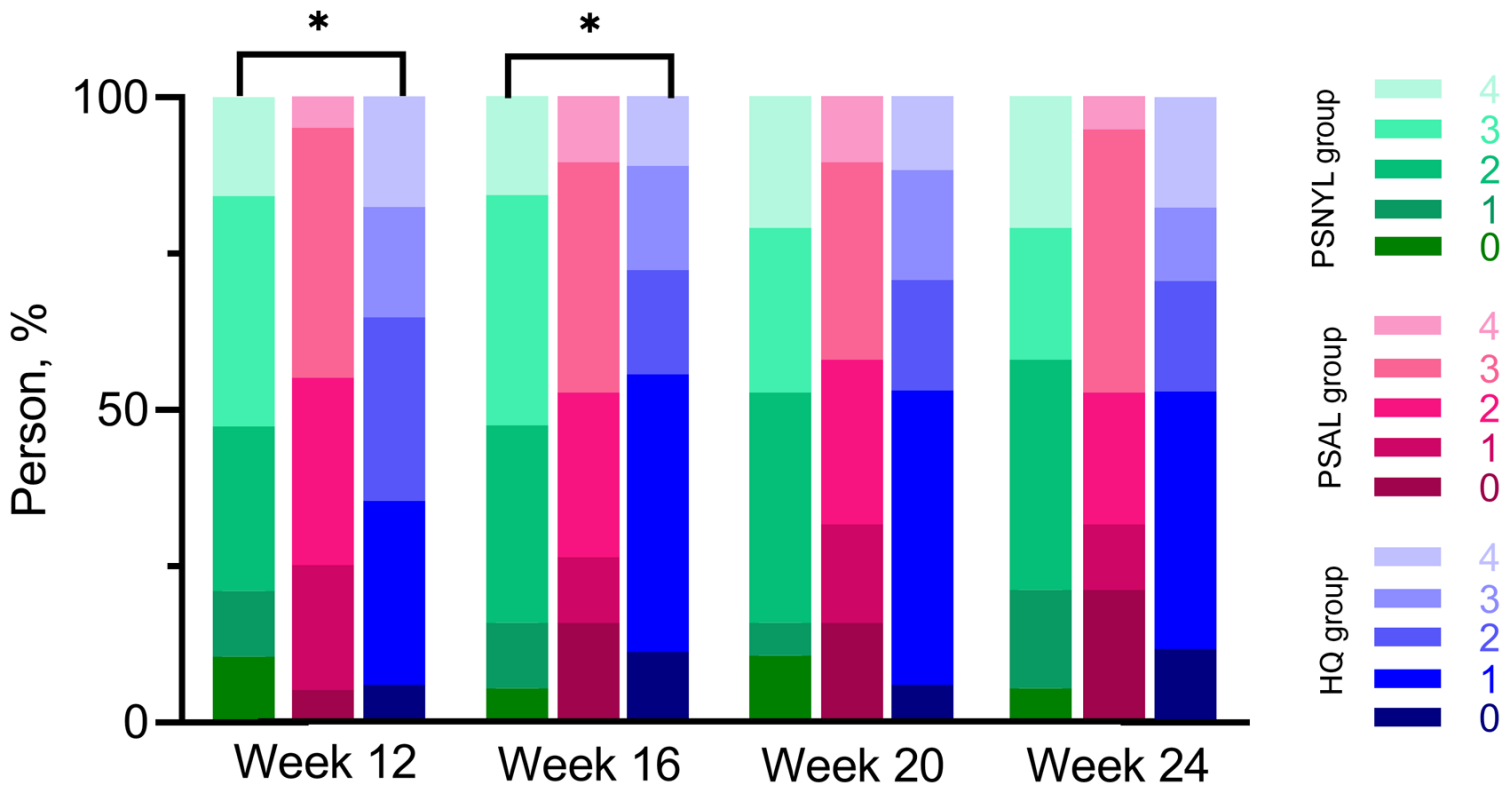
Patient assessment scores using a quartile grading scale. PSNYL, picosecond Nd:YAG laser (1,064 nm); PSAL, picosecond alexandrite laser (755 nm); HQ, hydroquinone (2%). Quartile Grading Scale: 0 (no improvement or worsening), 1 (less than 25% improvement), 2 (25 to <50%improvement), 3 (50 to <75% improvement), and 4 (75 to 100% improvement). * *p* < 0.05. At each follow-up timepoint, the patient assessment score in the PSNYL group seemed higher than that in the PSAL group and HQ group; the score in the PSAL group seemed higher than the HQ group. However, significant differences were only found between PSNYL and HQ groups at weeks 12 and 16.

Within-group differences in patient assessment scores were not significant. The group-by-time interaction was significant between the three groups (*p* = 0.031). Although the group main effect was not significant (*p* = 0.057), the patient assessment score in the PSNYL group was 1.81 times higher than that in the PSAL group (OR = 1.81 [0.62, 5.28], *p* = 0.280) and 3.60 times higher than the HQ group (OR = 3.60 [1.26, 10.28], *p* = 0.017); the PSAL group had 1.99 times higher scores than the HQ group (OR = 1.99 [0.65, 6.08], *p* = 0.225). The trend in the comparison results was observed at each follow-up timepoint (Table 4), however, significant differences were only found between PSNYL and HQ groups at weeks 12 and 16 (Figure 6).

**Table 4:** Outcomes of Between-group Differences in patient assessment scores.

Evaluation time	PSNYL group vs. PSAL group		PSNYL group vs. HQ group			PSAL group vs. HQ Group
	OR (95% CI)	*p* value	OR (95% CI)	*p* value	OR (95% CI)	*p* value
Week 12	1.18 (0.39 to 3.63)	0.767	3.21 (1.03 to 10.02)	**0.045**	2.71 (0.84 to 8.76)	0.096
Week 16	1.80 (0.59 to 5.50)	0.299	3.31 (1.05 to 10.44)	**0.042**	1.83 (0.58 to 5.83)	0.305
Week 20	1.39 (0.48 to 4.03)	0.543	2.08 (0.67 to 6.45)	0.203	1.50 (0.49 to 4.54)	0.477
Week 24	1.41 (0.41 to 4.82)	0.586	2.68 (0.87 to 8.27)	0.087	1.90 (0.58 to 6.22)	0.286

PSNYL, picosecond Nd:YAG laser (1,064 nm); PSAL, picosecond alexandrite laser (755 nm); HQ, hydroquinone (2%); OR, odds ratio; CI, confidence interval.

*p* values in boldface indicate significance.

### Safety analysis

Recurrence and PIH were reported in all three groups, with no significant differences among the groups (Table 5). PIH resolved spontaneously within 1 to 6 months. Only one patient in the HQ group developed hypopigmentation and recovered after 3 months. This patient experienced hyperpigmentation, erythema, and itch at the same time. Erythema and itch were seen in a total of two patients in the HQ group and the symptoms subsided within 1 week. But one patient asked to withdraw from the study because of the discomfort and the other discontinued HQ therapy but continued with follow-up (Figure 1).

**Table 5:** Adverse or unanticipated events after treatment.

Adverse or unanticipated events	PSNYL group *n* = 20	PSAL group *n* = 20	HQ group *n* = 19	Between-group *p* value
Recurrence	1(5.0)	2(10.0)	1(5.3)	>0.999
PIH	1(5.0)	1(5.0)	1(5.3)	>0.999
Hypopigmentation	0	0	1(5.3)	0.322
Erythema and itching	0	0	2(10.5)	0.100
Total count	2(10.0)	3(15.0)	3(15.8)^#^	0.900

Data are *n*(%). PIH, post-inflammatory hyperpigmentation.

# One patient in the HQ group developed PIH, hypopigmentation and erythema and itching at the same time.

The means (SD) of patients’ pain scores in the PSNYL and PSAL groups were 4.7 (1.3) and 5.3 (1.5), respectively, with no significant difference. The median (IQR) duration of erythematous appearance after laser interventions was 1.3 (2.5) hours in the PSNYL group and 2.0 (2.6) hours in the PSAL group, with no significant between-group difference.

## Discussion

Our results demonstrated that PSNYL or PSAL could significantly reduce MASI scores in melasma patients with FSTs III-IV, just as topical 2% HQ cream did, suggesting that both Picos are effective in treating melasma. However, PSNYL had a significantly better improvement of the MASI score than PSAL which showed comparable efficacy as 2% HQ. The PSNYL group showed the highest patient assessment score, followed by the PSAL group and then the HQ group, although only the differences between PSNYL and HQ groups at weeks 12 and 16 were significant. Recurrence occurred in a total of 6.8% (4/59) of patients in all three groups, and one patient in each group developed PIH that subsided within 1–6 months. No hypopigmentation or erythema and pruritus were observed in the laser groups. Our data suggested that PSNYL or PSAL is an alternative to 2% HQ cream for melasma, whereas PSNYL is a better choice for patients with FSTs III and IV.

Our results were consistent with those described in other studies which also confirmed the efficacy of PSNYL (11, 14–17) and PSAL (5, 8–10, 18–20) for melasma. However, only two (14, 21) and three (5, 9, 22) of the studies were RCTs regarding non-fractional PSNYL and PSAL, respectively. This randomized controlled study aimed to provide reliable evidence-based interventions for the clinical treatment of melasma using Picos. Our data showed for the first time, to the best of our knowledge, that non-fractioned PSNYL may have better efficacy than non-fractioned PSAL for melasma patients with FSTs III-IV. We can only speculate that the difference in wavelength or pulse duration of the lasers resulted in a different outcome. Although epidermal hyperpigmentation is a characteristic of melasma, findings of a heterogenous distribution of melanophages under confocal and multiphoton microscopies (23) indicate that all melasma is of mixed type (24). The basement membrane in melasma has been observed to be disrupted, which facilitates the travel of melanocytes and melanin down into the dermis (24). Moreover, melasma is a disorder not only limited to melanocytes, dermal factors have key roles in the pathogenesis, such as solar elastosis, increased mast cells, and vascularization (25). The longer wavelength of 1,064 nm PSNYL is associated with less epidermal melanin absorption and deeper penetration in the dermis (26), which is probably one of the reasons why it was more effective than 755 nm PSAL. A split-face clinical trial demonstrated that 1,064 nm Q-switched Nd:YAG (QSNYL) and 755 nm Q-switched alexandrite laser (QSAL) were equally effective at improving moderate to severe facial melasma (27). In the nanosecond range of pulse duration, the interference of the photothermal effect may be one of the reasons that the advantage of wavelength in 1064 nm QSNYL relative to 755 nm QSAL did not show up. In addition, the pulse duration of PSNYL was 450 ps in this study, which was shorter than the 750 ps of PSAL. A shorter pulse duration contributes to a higher photomechanical effect on the target chromophore and may be more effective for the destruction of the target (28). Anyway, additional studies are needed to provide a more accurate understanding of the underlying mechanisms for the efficacy difference of Picos in treating melasma.

Previous studies have shown that PSNYL is effective for melasma in both fractional (11, 15) and non-fractional(14, 16, 17) modes, and we chose to conduct the study in the non-fractional mode, which is a more economical option for patients. This was the first demonstration that the MASI score declined significantly after just one treatment session of PSNYL compared to baseline (*p* < 0.001). Furthermore, PSNYL showed superior efficacy to PSAL after two sessions (*p* = 0.027) and superior efficacy to 2% HQ after three sessions (*p* = 0.003). This dominance persisted throughout the follow-up to the end (week 24). Perhaps it was our parameter settings that made the treatment achieve inspiring results. We referred to the parameters of another study using the same laser device in a non-fractional mode (17). Their settings with a spot size of 6 mm and fluence of 0.54–1.22 J/cm^2^ led to 100% (4/4) melasma pigments achieving 50–100% lightening (good and excellent improvements) after a total of 8 or 9 treatments of non-fractional PSNYL (17). A larger spot size of 7 mm was applied in our research and the fluence range was narrowed down to 0.75–0.90 J/cm^2^. The findings of the present study suggested that the above parameter settings of non-fractional PSNYL are suitable for replication for melasma individuals with FSTs III and IV.

A split-face trial comparing PSAL with and without diffractive lens array (DLA) in the treatment of melasma found that there was no significant difference between the two sides in both objective and subjective assessments (5). In addition, PSAL without DLA, namely the non-fractioned one, offered a comparable clinical outcome but less downtime, less treatment discomfort, and lower incidence of PIH than the fractioned one (5). Based on the above evidence, PSAL in non-fractional mode was utilized in the present study. We found one treatment of non-fractioned PSAL resulted in significant improvement in melasma (*p* < 0.001) and changes in MASI scores increased over the intervention period, which was in line with the results of a previous RCT (8). Another RCT demonstrated that PSAL with DLA showed comparable efficacy with TCC (10), the gold standard treatment of melasma. In this study, at parameter settings of the spot size of 6–8 mm and the fluence of 0.40–0.71 J/cm^2^, the improvement in MASI scores of non-fractional PSAL was comparable to that of 2% hydroquinone, no matter in the overall analysis ignoring the time effect (the comparison of group main effect) or in the analysis of each time point.

A 1-year prospective cohort study showed that after 3–5 sessions of PSAL therapy, the MASI continually and significantly declined until the one-year follow-up (18). Another two studies also demonstrated sustained improvement in melasma after the Picos treatment over a three-month follow-up (8, 10). And this phenomenon was reproduced in our study. Some believed that it was the reaction induced by the laser intervention that led to the maintenance of the outcome (18). However, the same change in the HQ group in the present study indicated that sun protection probably played a critical role in post-treatment maintenance. Direct sun exposure is one of the leading risk factors for melasma, reported by 84% of patients as a factor of clinical impairment (2). Application of broad-spectrum sunscreen throughout the pregnancy was found to be effective in the prevention of the development of melasma in pregnant women (2.7% incidence in the study vs. 53% in usual conditions) (29). Regular use of broad-spectrum sunscreen alone for 12 weeks significantly improved the MASI in 100 Indian melasma patients (30). Thus, strict photoprotection is an integral part of all treatments for melasma. During the participation period of this study, photoprotection education was delivered to each subject at every visit time to reinforce their self-awareness and behavioral measures regarding sun exposure. Perhaps establishing a control group of patients who applied sunscreen alone for melasma would better illustrate the reason for post-treatment maintenance, which is a limitation of our study.

Patients seemed to be more satisfied with the efficacy of the lasers than with 2% HQ in our study. Two patients experienced erythema and pruritus after topical application of 2% HQ. Although the symptoms completely resolved after a week of discontinuation, they were no longer willing to use HQ and one of them requested to withdraw from the study. PIH and hypopigmentation appeared simultaneously in the other patient and the dyschromia condition gradually subsided within 3 months. Daily use of HQ can lead to high rates of irritation and dyschromia can develop when applied to normal skin (7), making HQ unfriendly to melasma patients with poor homogeneity, as in the two patients mentioned above. Their lesions presented as numerous small spots and patches scattered over the whole face, so a targeted application can be difficult and normal skin was often involved. As a result, they experienced side effects affecting nearly the entire face, which seriously undermined their confidence in the treatment of melasma. For these melasma patients with FSTs III-IV who cannot tolerate HQ, our data suggest that Picos, especially PSNYL, could be an alternative.

PIH was transient and was recovered spontaneously in the current study. However, relapse remains to be a challenge for the treatment of melasma because a complete understanding of its complex pathogenesis with multiple factors has yet to be achieved. Neagu et al. (1) believed that no single therapy was universally efficacious for melasma and that a combination of double or triple therapies yielded better results compared with monotherapy. Theoretically, the combination therapy with multitarget effects on different pathological mechanisms could exert a synergistic effect and may reduce the recurrence of melasma. Results of several previous studies regarding Picos have supported a synergistic effect of combination therapy. Choi et al. (21) showed that a picosecond laser with dual wavelengths (1,064 and 595 nm) and 2% HQ combination therapy had superior efficacy to 2% HQ monotherapy at the end of the treatment stage. Chalermchai et al. (11) found that fractional PSNYL combined with 4% HQ showed a greater modified MASI (mMASI) reduction than 4% HQ alone at 1 month after the last therapy. Li et al. (31) demonstrated that fractional PSAL and topical tranexamic acid (TTA) combination therapy had an obvious advantage over PASL monotherapy in treating melasma at 1- and 3-month post-treatment. However, mMASI or hemi-MASI displayed an increasing trend in the later stages of follow-up in the two studies mentioned above (21, 31), suggesting a likelihood of melasma recurrence after a period of cessation of treatment. Moreover, a recent split-face study suggested that there was not a substantial advantage in the PSAL and 2% HQ combination therapy versus the 2% HQ monotherapy (22). Thus, whether combination therapy has benefits and in what specific domains, such as enhancing the therapeutic effect or extending the duration of the therapeutic outcome, are problems that still require scrutiny. In conclusion, additional research to address the problem of melasma recurrence is needed.

Our study had a few limitations. First, 15% (9/60) of subjects did not complete the entire seven evaluations. But 98.3% (59/60) of subjects were included in the analysis. Because GEE used in the data analysis can accommodate missing outcomes under the missing-at-random assumption. Second, a control group with sunscreen application alone was not established since the primary objective of this study was to compare the efficacy and safety of PSNYL, PSAL, and 2% HQ. Third, this study was conducted in northern China, where the extremely dry climate in autumn and winter may affect the participants’ skin condition and thus the outcome of the study.

Overall, we showed that the efficacy of non-fractional PSNYL (1,064 nm) was superior to non-fractional PSAL (755 nm), while the efficacy of non-fractional PSAL was not inferior to the effect of 2% HQ, thus Picos offering alternatives for melasma patients with FSTs III-IV. The safety profiles of PSNYL, PSAL, and 2% HQ were similar.

## Data availability statement

The original contributions presented in the study are included in the article/supplementary material, further inquiries can be directed to the corresponding authors.

## Ethics statement

The studies involving human participants were reviewed and approved by the Ethical Committee of Beijing Friendship Hospital, Capital Medical University (2021-P2-118-02). The patients/participants provided their written informed consent to participate in this study. Written informed consent was obtained from the individual(s) for the publication of any potentially identifiable images or data included in this article.

## Author contributions

SL and SS conducted the research. WZ and AT evaluated the primary outcomes based on standardized photographs. SL was in charge of data analysis, table and figure creation, and manuscript writing. BZ assisted SL. XM and LL proposed hypotheses, supervised the research, and revised the manuscript. All authors made meaningful contributions to the study and approved the submission of this manuscript.

## Funding

This study is supported by Capital’s Funds for Health Improvement and Research (CFH 2022–2-20212).

## Conflict of interest

The authors declare that the research was conducted in the absence of any commercial or financial relationships that could be construed as a potential conflict of interest.

## Publisher’s note

All claims expressed in this article are solely those of the authors and do not necessarily represent those of their affiliated organizations, or those of the publisher, the editors and the reviewers. Any product that may be evaluated in this article, or claim that may be made by its manufacturer, is not guaranteed or endorsed by the publisher.
